# Experimental Analysis of Fabrication Parameters in the Development of Microfluidic Paper-Based Analytical Devices (µPADs)

**DOI:** 10.3390/mi8040099

**Published:** 2017-03-25

**Authors:** Wilson Lee, Frank A. Gomez

**Affiliations:** Department of Chemistry and Biochemistry, California State University, Los Angeles, 5151 State University Drive, Los Angeles, CA 90032-8202, USA; wlee27@calstatela.edu

**Keywords:** microfluidic paper-based analytical devices (µPADs), fabrication, optimization, wax, point-of-care (POC)

## Abstract

Microfluidic paper-based analytical devices (µPADs) have emerged as viable multiplexable platforms with the potential to transcend existing analytical techniques in resource-limited settings. µPADs are fabricated by patterning hydrophobic materials on hydrophilic paper. Reproducibility in fabrication is essential in a myriad of applications and particularly, in the development of point-of-care (POC) diagnostic devices that utilize paper-based platforms. A critical step in fabrication involves the wax heating process that determines the channel dimensions and the depth at which hydrophobic wax material permeates paper to create barriers. In this paper, we assess µPAD viability by examining two fabrication parameters that affect wax ink spreading and permeation using a commercial heat press: temperature and time of heating. Analysis of the µPADs revealed that functional chips could be fabricated at temperatures between 143 and 215 °C and time of heating between 50 and 135 s, while non-functioning chips were obtained at temperatures between 76 and 140 °C and time of heating between 5 and 45 s. Wax ink spread and permeated paper consistently between 143 and 215 °C. Also shown is a simple three dimensional (3D) microfluidic channel fabricated in a single sheet of cellulose paper utilizing the fabrication conditions described herein. This work demonstrates that controlling the extent of wax printing in the fabrication process of a µPAD can yield versatile and interesting devices for use in both resource-rich and -limited settings.

## 1. Introduction

Since the original publication nearly 25 years ago documenting the first microelectromechanical (MEM) device, microfluidic technologies have proliferated into nearly all sectors of the chemical, biological medicinal, and agricultural industries. Early devices were comprised of glass or polymeric materials including poly(dimethylsiloxane) (PDMS), polymethyl methacrylate (PMMA), and polyvinyl chloride (PVC). It became apparent that these initial lab-on-chip (LOC) devices could be of particular value in the point-of-care (POC) diagnostic arena due to their performance characteristics (specificity, sensitivity, and high accuracy). Correspondingly, these devices were time-consuming to fabricate due to their complexity, difficulty in reproduction, and sometimes high cost to manufacture [1,2,3,4,5].

Microfluidic paper-based analytical devices (µPADs) were first introduced by Whitesides in 2007 and have emerged as a viable multiplexable platform with potential to complement existing analytical techniques and in resource-limited regions of the world [6,7]. µPADs offer a number of advantages as a platform for microfluidics including low cost and ability to laterally flow fluids by capillary action, thereby, easily distributing small volumes of sample into various regions of the device [8,9,10,11]. There are multiple processes that can be undertaken to fabricate µPADs, each of which has various advantages and disadvantages. Photolithography was introduced by Whitesides in a glucose assay and a myriad of µPAD fabrication methods have been created since then [12]. The channels are highly reliable, although photolithographic techniques frequently require a master mold from which to make castings [13].

Laser printers have been extensively used to cut channels from hydrophobic paper, wherein the paper is pressed between glass slides to fabricate µPADs. This simple process has allowed for more intricate channels comparable to the use of PDMS µPADs [14]. µPAD fabrication has been shown using wax dipping where an iron mold was used to deposit wax onto chromatography paper. This process is inexpensive but also requires a mold to deposit wax onto the paper in a pattern. Furthermore, the process lacked the ability to fabricate intricate designs [15]. µPADs can also be fabricated using permanent markers with or without the use of an *XY* Plotter to pattern hydrophobic barriers [16]. This process allowed for the design of intricate channel platforms while minimizing costs in fabrication.

Wax printing is a more advantageous process that allows for more intricate patterning and reproducibility in the fabrication of µPADs [17]. It requires the use of a wax printer where wax is stored in “ink” cartridges. The wax is heated within the printer and drops onto the paper through an internal printer mechanism. The heated wax cools on contact with the paper in a precise and reproducible pattern created earlier by a computer drawing program. This removes the need to pattern the designs by hand or the use of a template, and contributes to the increased rate of fabrication. With this method of µPAD fabrication, there are two factors that should be considered when designing, printing and fabricating: time and temperature of heating. The inherent nature of wax and how it interacts with paper requires consideration when designing a µPAD. Upon heating, wax spreads laterally (*x*- and *y*-direction) as well as permeates (*z*-direction) through the paper. Hence, the original computer designed graphics detailing where printing (wax was to be placed) was to occur is modified. Even miniscule changes in chip dimensions can have profound effects in fluid flow, potentially deeming the µPAD unusable. Incomplete melting of wax could lead to leaking of sample outside the confines of the µPAD, as the wax might not have permeated the surface of the paper enough to form the hydrophobic barriers. In a similar fashion, overheating a wax chip with intricately designed channels could lead to blocked channels, as the wax barriers might spread more than desired.

Herein, we describe the effect of temperature and time of heating on µPAD viability by examining changes in line width and depth of wax permeation using a commercial heat press. Also shown is the fabrication of a simple three dimensional (3D) microfluidic channel on paper that utilizes the correlation between temperature and time of heating and wax spreading and permeation in its design. This work demonstrates that varying the parameters involved in the printing of wax-based µPADs allows for the fabrication of intricate devices for a myriad of applications.

## 2. Materials and Methods

### 2.1. Materials

All designs within this study were printed on Whatman grade 1 cellulose chromatography paper (0.18 mm thickness) using a Colorqube 8580 Solid Ink color printer (Xerox, Norwalk, CT, USA). The wax in this system consists of multiple colors (cyan, magenta, yellow, black). The viability testing of all µPADs or line strokes were conducted with solutions of blue or red food dye (Kroger, Cincinnati, OH, USA) (1 drop/10 mL water). µPADs were heated on a Hotronix Auto-Open Clam heat press (Stahl’s, Sterling Heights, MI, USA). Measurements of wax spreading and permeation were taken with a Keyence VHX-700F digital microscope (Keyence, Itasca, IL, USA). A heat press was chosen as the source of heating, due to its reproducible time and temperature settings, as well as its ability to heat sheets of paper evenly on both sides.

### 2.2. Fabrication

Patterns were designed on the computer using Inkscape software (0.92x, The Inkscape Project, open-sourced). Shapes and lines can be modified and unified to become any completed design. Using this software, the stroke of the line and channel widths can be changed. After a sheet of µPADs is printed, it is placed onto a heat press and heated at varying temperatures for a desired time. Time and temperatures were both determined via the readout of the heat press controller board, and confirmed with a stopwatch and contactless thermometer, respectively.

**Figure 1:**
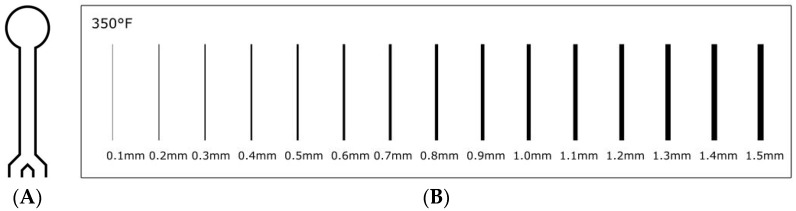
(**A**) Glucose assay microfluidic paper-based analytical device (µPAD) design and (**B**) strip of varying stroke-widths.

A µPAD design developed in an earlier glucose assay (Figure 1A) was used for the channel width optimization study [18]. The µPAD consisted of lines of 0.3 mm stroke width prior to heating. The study regarding changing line strokes and wax ink permeation utilized a strip of 0.1–1.5 mm lines. Here, an identical fabrication process was undertaken, but with a straight line design (Figure 1B). After printing and before heating, the channel widths and line thicknesses were verified using a microscope.

µPAD designs with 3D microfluidic channels were created with double sided printing. The two sides of the design were created using Inkscape software, where varying stroke widths (0.1–0.3 mm) were chosen based on their depth of permeation to create the 3D channel. The µPAD was heated at 176 °C and for 120 s.

## 3. Results and Discussion

### 3.1. Analysis of µPAD Channel Widths

**Figure 2:**
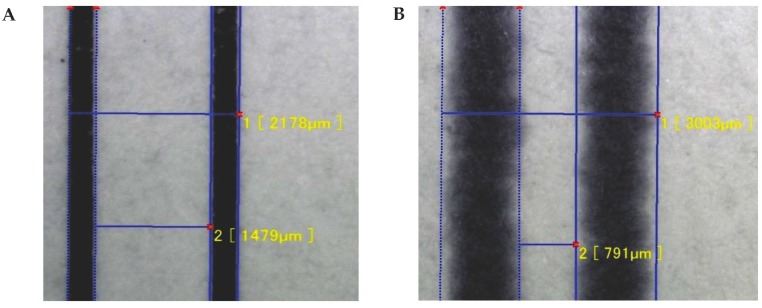
Images of (**A**) µPAD before heating and (**B**) µPAD after heating.

In this study, we adjusted two fabrication parameters (time and temperature of heating) to control the extent of wax spreading on and permeation into paper due to heating using a commercial heat press. While previous studies have examined a number of variables to assess wax behavior in paper, there has not been an exhaustive one involving heating time or temperature of heating [19,20]. To study these parameters, we fabricated a series of identical µPADs to determine the effect on the width of the channels. The µPADs were printed, heated, and cut into strips with the back surface left unobstructed. Measurements of widths before and after heating for the µPAD analyses were taken at 600× magnification. For the first µPAD design, the outer and inner widths of the rectangular analysis site are initially measured (Figure 2A) and measured again after heating (Figure 2B). The design of the chip allowed for easy and consistent analysis of the rectangular shaped site.

**Figure 3:**
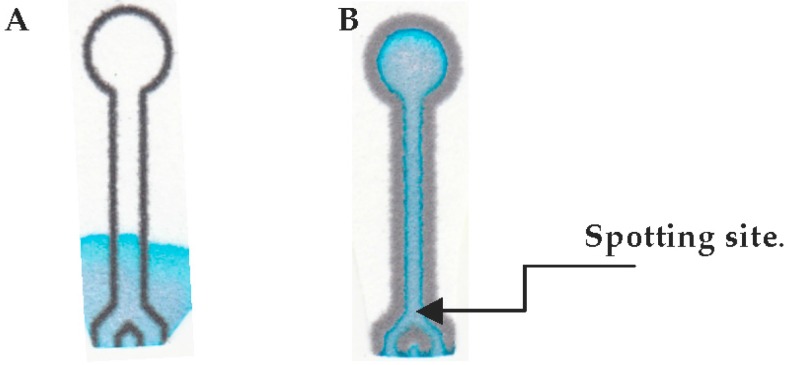
Images of a (**A**) non-viable and (**B**) viable µPAD.

After the channel width is measured, the µPADs were tested for viability upon varying the time and temperature of heating. Blue food dye (1 drop/10 mL water) was spotted onto the µPAD and sufficient time was allowed for it to absorb into the paper. Figure 3A shows a µPAD that is deemed non-viable as leakage of blue dye is observed outside the region marked by the wax barriers. The leaks occurred mainly on the back side of the device (opposite the printed side) because the wax did not permeate through the paper and was insufficient to form the hydrophobic barrier. Figure 3B is a viable µPAD since the blue dye is retained within the region marked, denoting the boundaries of the wax borders.

**Figure 4:**
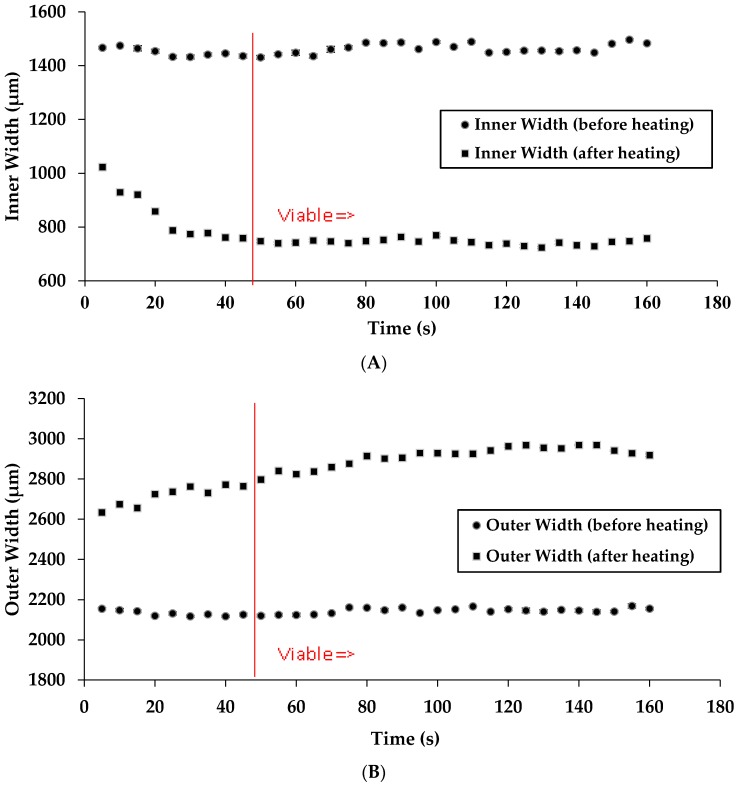
Experimental results of changes in the inner width (**A**) and outer width (**B**) versus time of heating at 176 °C.

We initially measured the inner and outer widths of the channels while keeping the temperature constant and changing the time of heating (Figure 4A,B). It was found that time of heating leads to a constant change in channel width even when reducing this time by more than one-half (120 to 48 s). Below ~48 s, there is not sufficient time for the wax to completely permeate through the paper as leaking was observed in the µPAD. Data points to the right of the red line in Figure 4A,B denote a viable chip fabrication time. It should be noted that in our previous laboratory research we had previously used a heating time of 120 s at 176 °C. These results reveal that a µPAD can be fabricated using a reduced time of heating than what was previously used. Data points to the right of the red line in Figure 4A,B denote a viable chip fabrication time.

**Figure 5:**
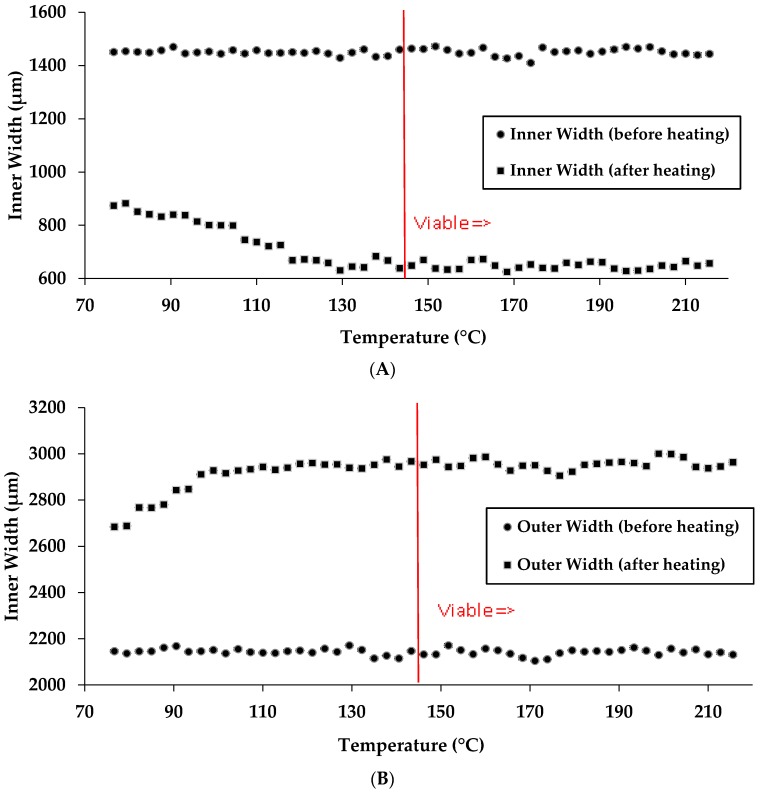
Experimental results of changes in the inner width (**A**) and outer width (**B**) versus temperature.

We then examined the effect of temperature of heating on the channel width of the µPADs. As previously stated, 176 °C is a sufficient temperature to create a viable barrier. It was found that lowering the temperature of heating to 143 °C while keeping time constant at 120 s still yields a viable µPAD. Increasing the temperature above 176 °C does not increase the line width, vis-à-vis no further spreading of the wax is observed (Figure 5A,B). At higher temperatures, the hot press is less consistent, as confirmed by a contactless thermometer reading. When the hot press is turned on, the metal heating plate is warmed up. At temperatures above 215 °C, the hot press surface temperature fluctuates 0.2%–0.3%, whereas, at lower temperatures, the instrument fluctuates less. Data points to the right of the red line in Figure 5A,B denote a viable chip fabrication time.

The time and temperature ranges used here are only a small window into potential combinations that create viable µPADs. That is, with lower temperatures, a higher time of heating could possibly produce a viable device. In the same sense, a lower time of heating with a higher temperature could also produce a viable µPAD. These combinations could be verified through further experimentation. Wax melting behavior may also vary depending on the type of paper used. Chromatography paper is manufactured and sold with different thicknesses, thus, with thicker paper, wax would likely spread less, due to its ability to permeate deeper into the paper.

**Figure 6:**
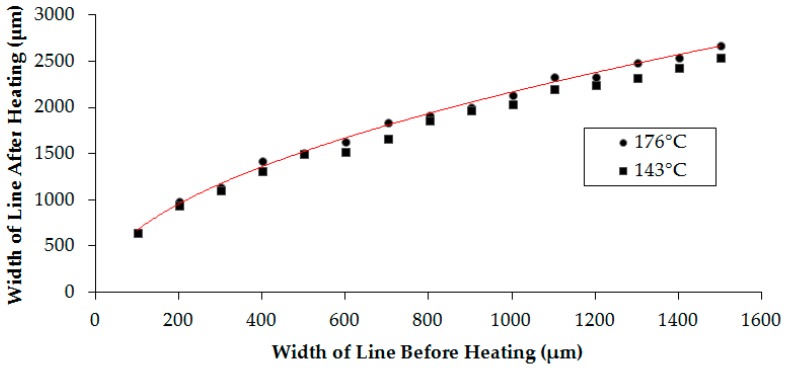
Experimental results of changes in the width of the line after heating versus width of the line before heating at 143 and 176 °C.

We then examined the effect of temperature on varying stroke widths. Here, a series of line strokes (Figure 1B) were printed in triplicate and measured before and after heating at two different temperatures (143 and 176 °C). Figure 6 shows the change in the width of the line before and after heating. As can be seen, there is little deviation at the two different temperatures. This result is expected as we described earlier that little change in the width of the channel is seen above 143 °C. A relationship between the width of the line before heating (*x*) and after (*y*) can be obtained (Equation (1)) that infers the final line width and, correspondingly, the width of the channel can be determined under these conditions. An arbitrary set of five line values was examined and the experimental values were compared to the theoretical values obtained using Equation (1) (Table 1). It was found that Equation (1) is a good predictor of line thickness after heating (<5% error).

*y* = 65.451*x*^0.5065^ (1)

Similarly, the relationship between channel width before and after heating can be assessed by use of Equation (2) (also determined at 176 °C). In this equation, the initial distance between these two printed lines is designated the “original channel width” (*O*_CW_), and the width of each line, “width 1” (*W*_1_) and “width 2”, (*W*_2_), respectively. The final distance between the two lines will be designated “final channel width” (*F*_CW_).

*F*_CW_ = *O*_CW_ − 0.5(*W*_1_ + *W*_2_) (2)

Any given line will spread a certain distance to the left and right side of its original print width midpoint, depending on the initial line stroke width. Note that *W*_1_ and *W*_2_ can differ, but in the case that *W*_1_ = *W*_2_, the function can be simplified further (Equation (3)). The function is valid, as the inner width of a channel decreases by the amount of spread of half of each line width.

*F*_CW_ = *O*_CW_ − (*W*_1_) (3)

The same line widths as those used in assessing Equation (1) were examined here. Each of the lines were placed in parallel with lines of identical widths, at arbitrary distances apart. It was determined with experimental data (Table 2), that Equation (3) can be used to predict the spreading of lines and with similar error (<5%).

**Figure 7:**
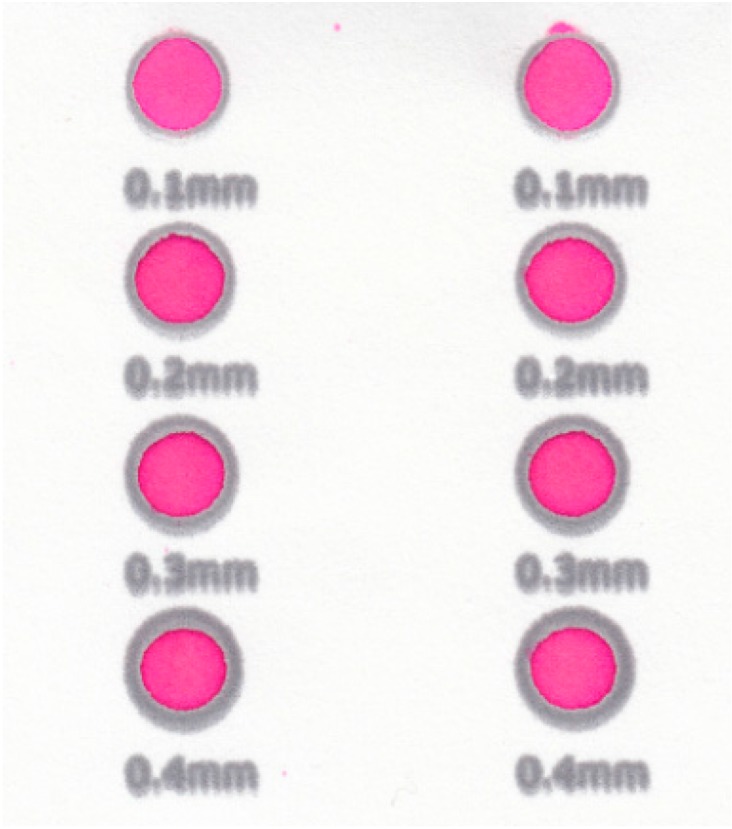
Examples of circles with varying stroke widths. The lettering is blurred due to the spreading of wax upon heating.

To examine the limit of dimension at which functional µPADs could be fabricated, different stroke widths were tested at a fixed time and temperature of heating (120 s, 176 °C) by printing circles of varying line strokes (Figure 7). A series of circles ranging in stroke widths from 0.1 to 0.4 mm was printed using Inkscape. An excess solution of red dye (10.0 µL) was spotted into each circle. A µPAD that retained dye within the circle was considered viable; a µPAD that did not retain dye was considered non-viable. The circles were run in triplicate.

### 3.2. Wax-Ink Permeation

**Figure 8:**
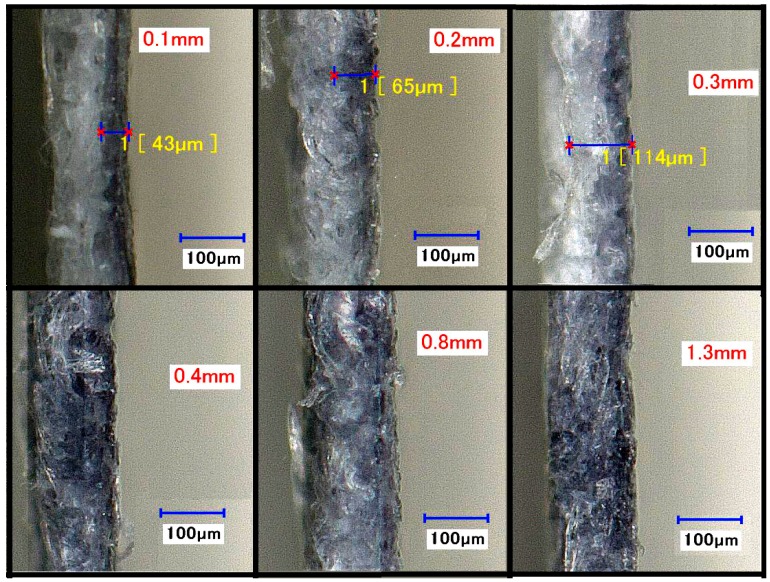
Microscopic cross section of varying stroke widths (top right in yellow) permeating at 176 °C (time of heating, 120 s).

The cross section of printed lines of varying stroke widths (red text) was examined under a digital microscope at 4000× magnification to assess how deep the wax permeated (yellow text) into the paper (Figure 8). It was found that increasing the stroke width (on the *x*–*y* axis) resulted in an increasing permeation of wax (on the *z* axis) at 176 °C (120 s heating). Wax permeated approximately 43 and 65 μm into the paper for stroke widths of 0.1 and 0.2 mm, respectively, whereas wax permeated completely through the paper for stroke widths greater than 0.3 mm, which permeated at approximately 114 μm. This increase in permeation is due to the greater amount of wax ink used in the printing. It is apparent from these results that the extent of wax permeation can be controlled by varying the width of the stroke widths. Previous experiments determined the spreading of wax on the *x*–*y* plane of the paper, but the experimental data here was focused on the depth at which each stroke width permeates [11].

**Figure 9:**
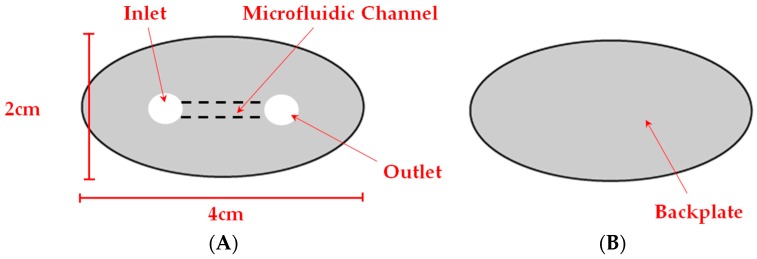
(**A**) Faceplate with low opacity and two blank spotting sites and (**B**) backplate with low opacity and no spotting sites.

**Figure 10:**
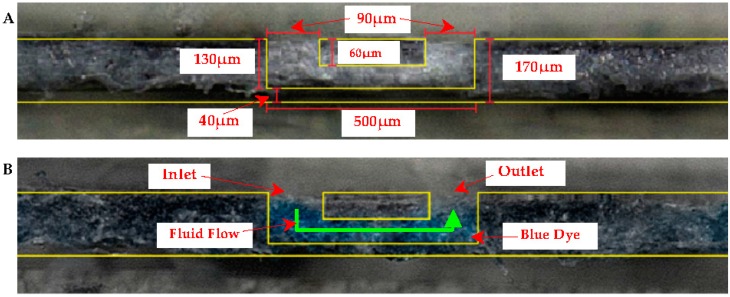
Inner channel cross section view (**A**) before dye was added and (**B**) after dye was added.

Using this technique, we explored the development of a 3D microfluidic channel within a µPAD. Its fabrication would require careful control of wax spreading and permeation. Figure 9A,B are a top-(faceplate) and bottom-view of the µPAD, respectively. The “faceplate” was designed with a lower opacity of ink such that the dye solution would not permeate through the hydrophobic barrier, and the barrier would not permeate through the paper in its entirety. A border was created with sufficient ink to contain the µPAD in an elliptical shape. A solution (2.0 µL) of blue dye was spotted into one of the circles, and was continually filled until the solution was visible in the other circle. Figure 10 shows microscopic cross section views of the faceplate region at 4000× magnification showing the µPAD before (A) and after dye is added (B) to the 3D microfluidic channel. Yellow lines are added to Figure 10A,B to emphasize the channel created within the paper. No leaking was observed in the device.

A 3D microfluidic channel can be created by printing wax on either side of the paper and heating the sheet on either side. The thinness of the chromatography paper allows for even heating on both sides, regardless of the orientation (face up or down). The fabrication of the 3D channel confirmed that it can effectively shield solutions from the environment and user thereby avoiding the potential for contamination, important in POC diagnostic devices. The use of thicker paper could yield more pronounced and intricate 3D channels for a variety of applications.

## 4. Conclusions

The current study has demonstrated that controlling the effects of temperature and time of heating of µPADs during the fabrication process can yield functional and reproducible chips, thereby allowing for their integration into a variety of applications. There is both a temperature and time of heating range at which a viable and consistent µPAD results. From a potential commercial aspect, this bodes well for the use of µPADs in a variety of settings and, in particular, the POC diagnostic area where there is great need for devices that are low cost, easily produced, sensitive, accurate, and reproducible. Another aspect of the study has revealed that at different initial stroke-widths of wax ink, the wax permeates the paper at different depths, effectively creating hydrophobic barriers at higher widths, but not at lower widths. A novel 3D microfluidic channel was produced by printing corresponding µPAD designs on both sides of a sheet of paper. We believe this technique can greatly simplify the fabrication of µPADs as well as improve their quality. Continued advances in device design due to fabrication optimization will embolden the use of paper-based platforms for understanding a wide range of global problems and in resource-limited settings.

## Figures and Tables

**Table 1:** A data set comparing experimental and theoretical values.

Original (µm) (Before Heating)	Experimental (µm) (After Heating)	Theoretical (µm) (After Heating)	Percent Error
236	1067	1041	2.50%
336	1297	1245	4.18%
456	1464	1454	0.69%
812	1941	1948	0.36%
985	2118	2148	1.40%

**Table 2:** A data set showing experimental and theoretical channel width changes.

*O*_CW_ (µm)	W_1_ (µm) Before/After Heating		Theoretical *F*_CW_ (µm)	Experimental *F*_CW_ (µm)	Percent Error
1500	236	1067	433	451	4.16%
1700	336	1297	403	430	6.70%
2200	456	1464	736	739	0.41%
2800	812	1941	859	844	1.75%
3800	985	2118	1682	1635	2.79%
